# The impact of accountability on local officials’ behavior in the pandemic prevention and control in China based on utility maximization

**DOI:** 10.1186/s41256-022-00268-w

**Published:** 2022-09-27

**Authors:** Shian Zeng, Chengdong Yi

**Affiliations:** School of Management Science and Engineering, Central University of Finance and Economics, Beijing, 102206 China

**Keywords:** Official accountability system, Pandemic prevention and control, Officials’ behavior, Utility maximization, Public health

## Abstract

**Background:**

The COVID-19 pandemic is a public health crisis and an inspection of national governance systems and crisis response capabilities of countries globally. China has adopted a tough accountability system for officials and has succeeded in containing the spread of the pandemic. This study aimed to assess the impact of accountability on local officials’ behavior in the pandemic prevention and control based on the official promotion tournament theory and utility maximization analysis framework.

**Methods:**

The panel data of 237 Chinese cities were extracted with local officials’ characteristics, confirmed cases, Baidu migration index, Baidu search index according to city names, and data were excluded with local officials’ relocation or sub-provincial cities between January 1, 2020 and May 5, 2020. Promotion gain and accountability cost were constructed by adopting promotion speed indicator, and the research hypotheses were assumed based on the utility maximization. It was the first time to apply the interaction model to empirically investigate the relationship between the promotion speed of local officials and the COVID-19 confirmed cases.

**Results:**

Our study showed that the promotion speed of provincial governors and mayors significantly affected the number of confirmed cases (β = − 11.615, *P* < 0.01). There was a significant interaction between the promotion speeds of provincial governors and mayors (β = − 2594.1, *P* < 0.01), indicating that they had a coordinated effect on the pandemic control. Additionally, mayors with different promotion speeds made a significant difference in controlling the imported cases and those who promoted faster better controlled the imported cases (β = − 0.841, *P* < 0.01). Mayors with full-time postgraduate education, titles, and majors in science and engineering had a better effect on controlling the number of confirmed cases.

**Conclusions:**

Our study provides evidence that the official accountability system has played an important role in containing the pandemic, which suggests that local officials motivated by the accountability system would respond to the pandemic actively for higher utility. Furthermore, provincial governors and mayors have played a coordinated effect in pandemic control. The above evidences reveal that implementing the official accountability system could improve the government’s emergency management capability and the efficiency of pandemic control. Therefore, adopting a strict accountability system could be effective in pandemic containment globally, especially in centralized countries.

## Introduction

The Corona Virus Disease 2019 (COVID-19) pandemic has challenged the national capacity of all countries in the world, and it is also a “touchstone” for testing their capabilities of crisis management [1]. As for the COVID-19 pandemic prevention and control, Chinese governments and officials have played an important role in the national governance system and public health emergency management [2]. Since the outbreak of COVID-19 in China, the central government has taken a series of effective measures to contain the pandemic, including large-scale quarantines, travel restrictions, and the isolation and monitoring of suspected cases [3]. Although virus mutation and importation of exogenous cases have led to several small-scale outbreaks rebounding in some areas of China, China has timely controlled the pandemic under the scientific guidance of the general strategy against imported cases and the rebound in indigenous cases with the dynamic zero COVID-19 policy [4].

In China, the governments possess the primary administrative and social resources, and their behaviors directly affect the state of affairs and the outcome of an incident. He et al. [5] think that many of the poor outcomes of crisis management could be largely attributed to the inefficiencies of bureaucracies. Allocating government officials to crisis management is critical to collaborative governance because dealing with emergencies, uncertainties, and complex situations requires officials to take prudent and creative strategies [6, 7]. To verify the effect of accountability on officials’ behavior in the pandemic prevention and control, the principal-agent relationship between local and central governments according to the official promotion tournament theory is applied [8–11]. In this study, we believe that the implementation of the official accountability system reinforces the rationality of this relationship. In China, local officials are in a closed internal labor market. Once removed or dismissed, they would face a huge drop inside and outside their careers [11]. Therefore, as for local officials, the cost of being held accountable for ineffective containment of the pandemic is enormous.

Some researchers have questioned the validity of the official promotion tournament theory in terms of political networks of officials and empirical data. They think that even if there is an association between the economic growth and the probability of official promotion, the reason behind this association is that officials with better political network relations are more likely to be assigned to places where they can perform better [12–15]. Furthermore, they hold that the tenure of local officials is not fixed and varies widely, making it difficult to identify the net effect of individuals on the economic growth [16]. In this study, two concerns that could affect the validity of the conclusions are avoided. First, we believe that the political network ties possessed by local officials could be ignored in the COVID-19 pandemic prevention and control. On the one hand, China has established a leadership team to respond to the pandemic, which is headed by the Premier Minister and reports directly to the Standing Committee of the Political Bureau of the Communist Party of China Central Committee. On the other hand, the COVID-19 outbreaks in regions are random events, and local officials with strong political network ties are unlikely to be pre-appointed in places where the pandemic is less likely to occur. Second, this study excluded the data of officials’ relocation during the study period to ensure that the effect of local officials’ behavior on the pandemic prevention and control is not interfered by other officials.

In this study, the impact of the personal capacity of local officials on the pandemic prevention and control was also investigated. Some researchers have used education as a proxy variable for capability, but this ignores the interaction between education and capability [17]. Relevant studies have shown that officials’ promotion experience reflects their comprehensive capability [18–20], and the promotion speed indicator can be used to measure officials’ capability. Therefore, the purpose of this study is to assess the impact of the accountability on the local officials’ behavior in the pandemic prevention and control by constructing the promotion gain and accountability cost with the promotion speed indicator.

## Methods

### Theoretical analysis and research hypothesis

Suppose that local officials have two choices in preventing and controlling the pandemic: *c* ∈ {0,1}, where *c* = 0 means local officials choose to prevent and control the pandemic actively, and *c* = 1 means local officials choose to prevent and control the pandemic passively. The utility of local officials depends on the promotion gain and accountability cost after they have a choice, and the behavior of local officials depends on the utility with the different choices. There is little difference in promotion gain for local officials who actively or passively prevent and control the pandemic because the central government hardly gives extra rewards to local officials who actively prevent and control the pandemic. For a responsible government, ensuring public interests is a basic requirement. According to Bai and Kung [21], and Jin and Shen[22], the promotion gain for local officials is set as follows:

1 $$\pi = \alpha + \ln pro\_speed$$

*α* is the intercept term, and *pro_speed* is the official promotion speed, which is calculated as the reciprocal of the officials’ age when they obtain a full departmental or ministerial rank position. In 1982, China abolished the lifetime system for leading cadres, clearly stipulated the retirement age for officials, and imposed strict age restrictions on the selection of officials. Therefore, local officials who promote faster have better career prospects before retirement, thus having relatively greater promotion motivation [22].

The Chinese central government usually uses the number of confirmed cases to determine whether to hold local officials accountable in the COVID-19 pandemic prevention and control. The higher the number of confirmed cases are, the higher the probability of local officials being held accountable is. Assume that the probability of local officials being held accountable is *p*, and the number of confirmed cases is *confirmed*, then the relationship between *p* and *confirmed* is *p* = λ*confirmed, *λ* > 0. In addition, it is assumed that the number of confirmed cases could be reduced when local officials actively prevent and control the pandemic, so the number of confirmed cases is *confirmed* = *β*_0_ + *β*_1_*c, *β*_0_ > *0*, *β*_1_ > 0. The probability of promotion for local officials is generally low, and local officials being held accountable usually means losing prospect for career promotion. Therefore, the cost of accountability for local officials is *κ*_0_ + *κ*_1_*pro_speed, and *κ*_1_ is an infinite positive number. For local officials who are not held accountable, the cost of accountability is *κ*_0_. By contrast, for local officials who are held accountable, the cost of accountability is *κ*_0_ + *κ*_1_. Since *κ*_1_ is an infinite positive number, *κ*_0_ can be considered as zero compared to *κ*_0_ + *κ*_1_. The total utility is calculated as follows:

2 $$\max U(c) = \alpha + \ln pro\_speed - [\lambda (\beta_{0} + \beta_{1} c)(\kappa_{0} + \kappa_{1} pro\_speed) + (1 - \lambda \beta_{0} - \lambda \beta_{1} c)\kappa_{0} ]$$

The behavior of local officials in the pandemic prevention and control depends on the difference between *U*(*c* = 0) and *U*(*c* = 1). If Δ*U* = *U*(*c* = 0) − *U*(*c* = 1) > 0, it indicates that local officials could obtain higher utility by positively preventing and controlling the pandemic. The mathematical expression of Δ*U* is:

3 $$\Delta U = \lambda \beta_{1} \kappa_{1} pro\_speed$$

*λ*, *β*_1_, *κ*_1_ and *pro_speed* are all positive, so Δ*U* is greater than zero. Therefore, local officials could obtain higher utility by positively preventing and controlling the pandemic than passively. Under the principle of utility maximization, local officials choose to prevent and control the pandemic actively. The first-order derivation of ∆*U* of Eq. (3) to *pro_speed* is:

4 $$\frac{\partial \Delta U}{{\partial pro\_speed}} = \lambda \beta_{1} \kappa_{1}$$

According to Eq. (4), ∆*U*’s first-order derivation to *pro_speed* is greater than zero, which indicates that the difference in local officials’ utility between the active and passive pandemic prevention and control becomes larger as the promotion speed increases. This indicates that officials with faster promotion speed could gain higher utility by behaving actively in the pandemic prevention and control. Therefore, this study assumes the first hypothesis:

#### H1

Under the utility maximization, officials who promote faster would be more active in the pandemic prevention and control, so they would have a better effect on controlling the number of confirmed cases.

Moreover, Lin [18], and Zang [23], consider that the characteristics of officials, such as full-time postgraduate education, titles, and type of undergraduate major, have a significant impact on the promotion speed. Besides, the different characteristics mean that officials’ experience and cognition are different, thus leading to the differences in behavior. Therefore, the second hypothesis is:

#### H2

There would be an interaction between the official characteristics and the promotion speed, and officials with different characteristics have a different effect on controlling the number of confirmed cases.

**Fig. 1:**
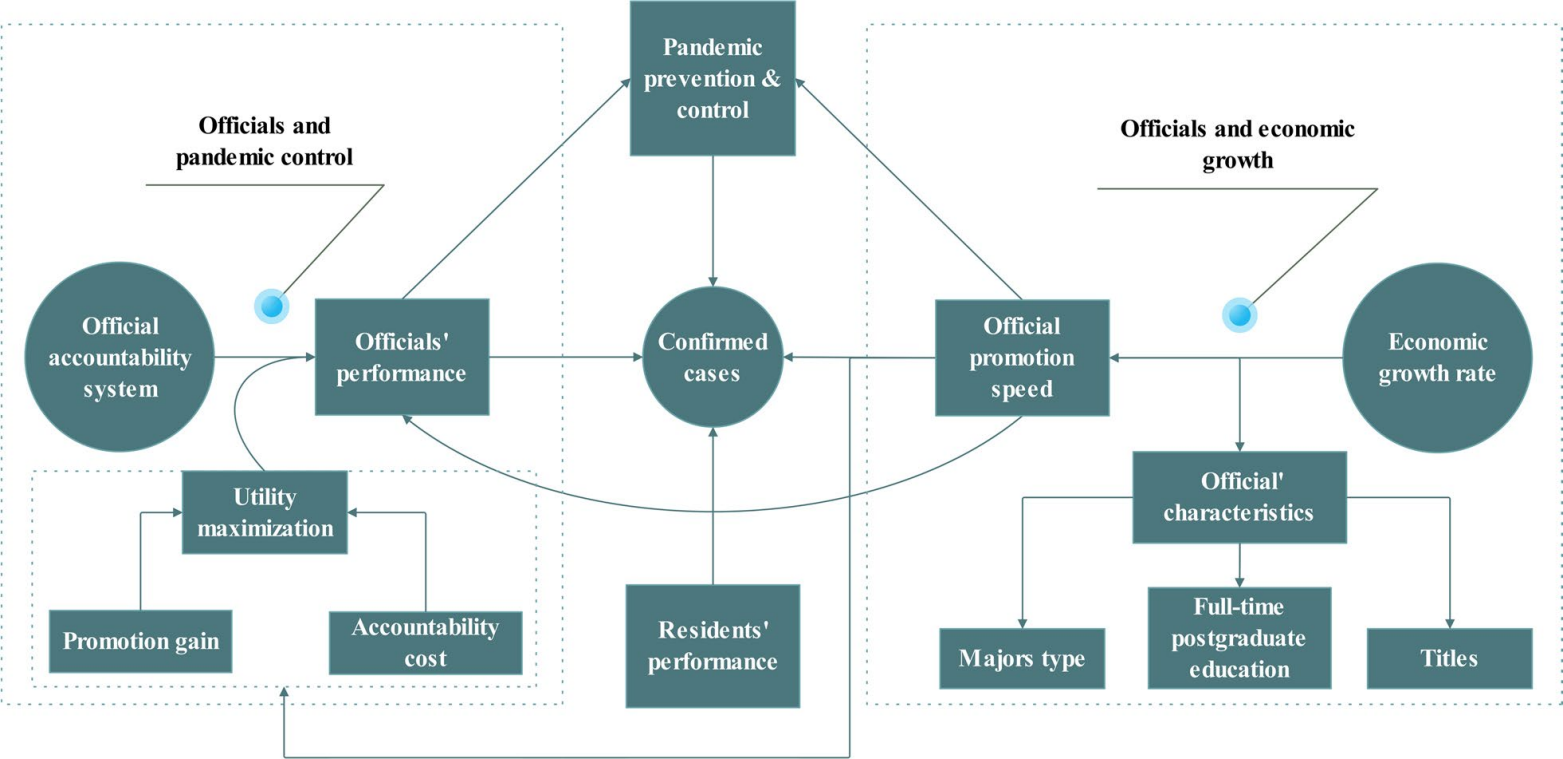
Conceptual model and analytical framework

Finally, some researchers have studied the effect of officials’ age on environmental policy implementation and environmental pollution improvement [24–26]. Jin and Shen [22] use the age of local officials as a core variable to construct the utility of local officials. Wang and Xu [20] think that younger officials have better career prospects and greater motivation for promotion. Thus, motivated by promotions, young officials would actively prevent and control the pandemic. Therefore, this study replaces the promotion speed variable with the real age of local officials for robustness testing. The conceptual model and analytical framework are shown  in Fig. 1.

### Variables and models

In order to identify the local officials’ effects on the pandemic control, this paper used the interaction model to study. In terms of control variables, this paper used the method of dummy variables to control the spatial differences of confirmed cases. In addition, this paper controlled the differences of total population, population mobility and residents’ awareness of epidemic prevention and control among cities. The specific model is set as follows:

5 $$\begin{aligned} \ln confirmed_{it} & = \beta_{0} + \beta_{1} mayor\_prorate_{it} + \beta_{2} wh\_emigraindex_{it} + \beta_{3} governor\_prorate_{it} \\ & \quad + \beta_{4} mayor\_emigraindex_{it} + \beta_{5} governor\_mayor_{it} + \beta X_{it} + t + \varepsilon_{it} \\ \end{aligned}$$

The core explanatory variables included *mayor_prorate*, *governor_prorate*, *wh_emigraindex*, *mayor_emigraindex*, and *governor_mayor*. *X* denotes the set of control variables, including *lnresident_aware*, *lnpopulation*, *migraindex*, *surrounding*, and *hubei_city*. *t* denotes the time trend variable, and *ε* denotes the disturbance term. The reasons for selecting the above variables for the study are shown in “Appendix”.

In the Eq. (5), the partial effect of the *mayor_prorate* on the *lnconfirmed* (keeping all other variables constant) is:

6 $$\mathop {effect}\limits^{ \wedge } = \frac{{\Delta \ln confirmed_{it} }}{{\Delta mayor\_prorate_{it} }} = \beta_{1} + \beta_{4} wh\_emigraindex_{it} + \beta_{5} governor\_prorate_{it}$$

To make the regression coefficient *β*_1_ meaningful and alleviate the multi-collinearity of variables, this study constructed the Eq. (7) by centering the interaction variables of the Eq. (5). Moreover, to further validate Hypothesis 2, a set of interaction variables for mayor characteristics (*major*, *title*, *full_time*) and the *mayor_prorate* variable were added to the Eq. (7), including interaction variables for *mayor_major*, *mayor_title*, and *mayor_full_time*. The Eq. (7) is specified as follows:

7 $$\begin{aligned} \ln confirmed_{it} & = \beta_{0} + \delta_{1} mayor\_prorate_{it} + \delta_{2} wh\_emigraindex_{it} + \delta_{3} governor\_prorate_{it} \\ & \quad + \delta_{4} (mayor\_prorate_{it} - \mu_{1} )(wh\_emigraindex_{it} - \mu_{2} ) \\ & \quad + \delta_{5} (mayor\_prorate_{it} - \mu_{1} )(governor\_prorate_{it} - \mu_{3} ) \\ & \quad + \delta_{j} \sum\limits_{j = 6}^{8} {mayor\_char_{it} } + \beta X_{it} + t + \varepsilon_{it} \\ \end{aligned}$$

*μ*_1_, *μ*_2_, and *μ*_3_ are the average values of the variables of *mayor_prorate*, *wh_emigraindex*, and *governor_prorate*, respectively. Moreover, since the explained variables in the Eq. (5) and Eq. (7) are logarithmic, when the coefficient of the explanatory variable is semi-elastic, and the absolute value is large, the estimation effect can be calculated more accurately by using the Eq. (8). Taking the *mayor_prorate* variable as an example, the effect on *lnconfirmed* is:

8 $${\%}\Delta \ln confirmed = 100 \times \left[ {\exp (\mathop {effect}\limits^{ \wedge } \times \Delta mayor\_prorate) - 1} \right]$$

### Data collection and processing

The data of confirmed cases were obtained from the official websites of China’s provincial and municipal health commissions. The data of officials’ characteristics were collected from portal websites of provincial and municipal governments and search engines such as Baidu. Baidu search index data came from the Baidu index platform. Population movement data came from the Baidu migration data platform.

The specific procedures of data processing were as follows: (i) data on confirmed cases was collected from January 1 to May 5, 2020 for more than 300 prefectures in China, covering 22 provinces, 4 autonomous regions, and 4 municipalities directly under the central government. Hong Kong, Macau, Taiwan and Tibet were excluded; (ii) the resume information of government officials was extracted from the portals of provincial and municipal governments as well as search platforms such as Baidu, and then screened and sorted; (iii) Baidu migration data and Baidu search index data were extracted from Baidu migration data platform and Baidu index platform, respectively; (iv) the above data was matched according to city names. Data for officials' relocation during the study period and sub-provincial cities were excluded.

### Data analysis

The data collected in this study covered 237 prefecture-level cities from January 1, 2020 to May 5, 2020. To exclude the interference of other officials, this paper excluded the data sample of officials’ relocation during the study period. In addition, because the mayors of sub-provincial cities in China have higher administrative levels than the mayors of prefecture-level cities, this paper excluded the data sample of sub-provincial cities. The final data sample accounted for 80.89% of the total sample. Moreover, the data was a long balanced panel, so there may be heteroscedasticity and autocorrelation in the disturbance terms. Therefore, this study used the panel-corrected standard error (PCSE) and feasible generalized least squares (FGLS) methods to deal with this problem. Finally, the core explanatory variable promotion speed did not vary over time, which implies that city fixed effects cannot be controlled in the model. Therefore, this paper added effective control variables to the model to mitigate the potential omitted variable problem. The analyses were conducted using Stata version 16.1.

## Results

### Descriptive statistics and demographic analysis

Table 1 reported the descriptions and descriptive statistical analysis of variables. Table 2 reported the demographic analysis of officials. A total of 267 officials were included in the study, of whom 237 were mayors of prefecture-level cities, 4 were mayors of municipalities directly under the central government, and 26 were provincial governors (including four autonomous regions governors). The proportion of prefecture-level city mayors who were male was 93.67%, and the proportion of those aged 50 years or older was 70.46%. The percentage of those with the titles of associate professor, economist, and engineer and above was 14.35%. In terms of education, the percentage of those whose undergraduate major type was liberal arts was 75.11%, and the percentage of those with full-time postgraduate degrees was 15.61%. The proportion of governors who were male was 90.00%. All were over 50 years old, 73.33% were between 55 and 60 years old, and more than half possessed titles (53.33%). In terms of education, the percentage of those whose undergraduate major type was liberal arts was 63.33%, and the percentage of those with full-time postgraduate degrees was 30.00%.

**Table 1:** Descriptions and descriptive statistical analysis of variables

Variables	Descriptions	(1)	(2)	(3)	(4)	(5)
		Sample size	Mean	Min	Max	SD
*lnconfirmed*	Number of confirmed cases is counted by logarithms	29,862	2.5520	0.0000	8.1660	1.7400
*mayor_prorate*	Mayor’s promotion speed	29,862	0.0192	0.0172	0.0286	0.0015
*mayor_age*	Mayor’s real age	29,862	52.2620	35.0000	58.0000	3.7180
*governor_prorate*	Provincial governor’s promotion speed	29,862	0.0175	0.0152	0.0300	0.0027
*governor_age*	Provincial governor’s real age	29,862	59.0930	55.0000	66.0000	2.7110
*wh_emigraindex*	Baidu migration index for Wuhan	29,862	0.0061	0.0000	1.8840	0.0484
*mayor_emigraindex*	Interaction between mayor’s promotion speed and Baidu migration index for Wuhan	29,862	0.0001	0.0000	0.0355	0.0009
*governor_mayor*	Interaction between mayor’s promotion speed and provincial governor’s promotion speed	29,862	0.0003	0.0003	0.0007	0.0001
*lnresident_aware*	Residents’ awareness of epidemic prevention and control	29,862	4.2720	0.0000	7.3010	2.1330
*lnpopulation*	Number of population is counted by logarithms	29,862	4.6920	2.7670	6.3310	0.6340
*migraindex*	Baidu migration scale index	29,862	0.7890	0.0095	9.5230	0.8200
*hubei_city*	Is the city in Hubei Province (Yes = 1)	29,862	0.0422	0.0000	1.0000	0.2010
*surrounding*	Is the city in the surrounding provinces of Hubei Province (Yes = 1)	29,862	0.2660	0.0000	1.0000	0.4420
*mayor_major*	Interaction between mayor’s promotion speed and undergraduate major type	29,862	0.0144	0.0000	0.0286	0.0084
*mayor_full_time*	Interaction between mayor’s promotion speed and postgraduate degree type	29,862	0.0030	0.0000	0.0227	0.0071
*mayor_title*	Interaction between mayor’s promotion speed and title	29,862	0.0028	0.0000	0.0217	0.0067
*major*	Is the mayor’s undergraduate major in liberal arts? (Yes = 1)	29,862	0.7510	0.0000	1.0000	0.4320
*full_time*	Is the mayor’s postgraduate degree full-time? (Yes = 1)	29,862	0.1560	0.0000	1.0000	0.3630
*title*	Does the mayor have a title? (Yes = 1)	29,862	0.1430	0.0000	1.0000	0.3510

**Table 2:** Demographic attributes of the officials

Position	Attributes	Distribution	Frequency	Percent (%)
Mayor	Gender	Male	222	93.67
		Female	15	6.33
	Age	30–40 years	1	0.42
		41–50 years	69	29.11
		Above 50 years	167	70.46
	Undergraduate major type	Science and engineering	59	24.89
		Liberal arts	178	75.11
	Postgraduate degree type	Full-time postgraduate	37	15.61
		Part-time postgraduate	200	84.39
	Title	Have	34	14.35
		Not have	203	85.65
Governor	Gender	Male	27	90.00
		Female	3	10.00
	Age	55–60 years	22	73.33
		61–66 years	8	26.67
	Undergraduate major type	Science and engineering	11	36.67
		Liberal arts	19	63.33
	Postgraduate degree type	Full-time postgraduate	9	30.00
		Part-time postgraduate	21	70.00
	Title	Have	16	53.33
		Not have	14	46.67

Mayor (prefecture level city), Governor (including the governors of provinces, autonomous regions, and municipalities directly under the central government). The mayor’s demographic analysis does not include officials’ relocation and sub-provincial cities. The governors demographic analysis does not include Hong Kong, Macau, Taiwan, and Tibet

### Mayor’s effect on the pandemic control

Table 3 shows the test results of the pandemic control effect of local officials. It can be seen from the results of column (1) in Table 3 that the promotion speed of the mayor and the promotion speed of the provincial governor are not significant. The results of column (2) show that the regression coefficients of the variables are the same as those from column (1), but the standard errors are obviously smaller, which leads to an increase in the statistical significance of the explanatory variables, such as the promotion speed of mayor and provincial governor are both significant at the level of 1%. The results of column (4) show that the promotion speed of the mayor and provincial governor is significantly positive at the 1% level, which is contrary to the results of columns (1) and (2). However, due to the interaction model used in this paper, it can be seen from Eq. (6) that the provincial governor’s promotion speed and the population inflow from Wuhan city could affect the mayor’s control effect on confirmed cases. To better explain the meaning of the regression coefficients of the variables, columns (3) and (5) show the results after centering the interaction terms. Therefore, the results from columns (3) to (5) are mainly reported in the following of this paper, and the results from columns (1) to (2) for reference.

**Table 3:** The official promotion speed and the pandemic prevention and control test

Variables/counterpart questions	(1)	(2)	(3)	(4)	(5)	(6)
	OLS	PCSE	PCSE_para	FGLS	FGLS_para	FGLS_para*
Mayor’s promotion speed	− 74.449	− 74.449***	− 13.975***	41.129***	− 12.623***	− 11.615***
	(150.589)	(25.943)	(1.598)	(0.739)	(0.156)	(0.215)
Provincial governor’s promotion speed	− 76.523	− 76.523**	5.961*	65.962***	9.930***	7.508***
	(171.503)	(30.200)	(3.275)	(0.997)	(0.396)	(0.445)
Baidu migration index for Wuhan	42.971***	42.971***	− 3.589***	7.637***	− 0.838***	− 0.841***
	(4.350)	(3.280)	(0.441)	(0.052)	(0.006)	(0.005)
Interaction between mayor’s promotion speed and Baidu migration index for Wuhan	− 2420.0***	− 2420.0***		− 440.5***		
	(216.834)	(173.096)		(2.949)		
Interaction between mayor’s promotion speed and provincial governor’s promotion speed	4287.262	4287.262***		− 2912.3***		
	(8448.69)	(1540.324)		(42.158)		
Residents’ awareness of epidemic prevention and control	0.526***	0.526***	0.526***	0.112***	0.112***	0.111***
	(0.014)	(0.015)	(0.015)	(0.00009)	(0.00009)	(0.00008)
Baidu migration scale index	0.091**	0.091***	0.091***	− 0.059***	− 0.059***	− 0.060***
	(0.036)	(0.019)	(0.019)	(0.0002)	(0.0002)	(0.0002)
Number of population is counted by logarithms	0.401***	0.401***	0.401***	0.605***	0.605***	0.594***
	(0.068)	(0.021)	(0.021)	(0.006)	(0.006)	(0.008)
Is the city in the surrounding provinces of Hubei Province (Yes = 1)	0.760***	0.760***	0.760***	0.727***	0.727***	0.716***
	(0.094)	(0.032)	(0.032)	(0.007)	(0.007)	(0.009)
Is the city in Hubei Province (Yes = 1)	4.009***	4.009***	4.009***	3.400***	3.400***	3.380***
	(0.709)	(0.124)	(0.124)	(0.032)	(0.032)	(0.043)
Time trend	0.006***	0.006***	0.006***	0.022***	0.022***	0.022***
	(0.0005)	(0.0009)	(0.0009)	(0.0002)	(0.0002)	(0.0002)
Centering the interaction between mayor’s promotion speed and Baidu migration index for Wuhan			− 2420.1***		− 440.4***	− 441.9***
			(173.101)		(2.952)	(2.309)
Centering the interaction between mayor’s promotion speed and provincial governor’s promotion speed			4287.6***		− 2912.5***	− 2594.1***
			(1540.386)		(42.162)	(65.508)
Interaction between mayor’s promotion speed and undergraduate major type						− 0.947***
						(0.025)
Interaction between mayor’s promotion speed and graduate degree type						− 0.629***
						(0.033)
Interaction between mayor’s promotion speed and title						− 6.671***
						(0.095)
_cons	− 1.032	− 1.032*	− 2.196***	− 3.490***	− 2.455***	− 2.347***
	(3.044)	(0.581)	(0.138)	(0.0358)	(0.0307)	(0.038)
*N*	29,862	29,862	29,862	29,862	29,862	29,862
*R*^2^	0.779	0.779	0.779			

Columns (1), (2), and (4) are the results of using Eq. (5). Column (1) shows the results of the OLS method controlling the time effects and robust standard errors of city clustering. Column (2) shows the results of the PCSE method when inter-group heteroskedasticity and inter-group contemporaneous correlation in the disturbance term. Column (4) shows the result of the comprehensive FGLS method, i.e., the intra-group autocorrelation of the disturbance term is considered based on the PCSE method. Columns (3) and (5) are the results of centering the interaction variables based on Eq. (5). Column (6) shows the results of Eq. (7) using the comprehensive FGLS method. Standard errors are reported in parentheses

*p < 0.1; **p < 0.05; ***p < 0.01

The results of columns (3) and (5) in Table 3 show that the mayor’s promotion speed has a negative effect on confirmed cases and is significant at the 1% level (β = − 13.975, p < 0.01; β = − 12.623, p < 0.01) when the provincial governor’s promotion speed and Wuhan’s Baidu migration index are averaged. Due to the large absolute value of the regression coefficients, using the regression coefficients to approximate the mayor’s pandemic control effect would result in a bias, so Eq. (8) was used for the calculation. As shown in Table 1, the minimum, mean, and maximum values of the promotion speed of mayors are 0.017, 0.019, and 0.029, respectively, corresponding to the ages of 59, 53, and 34 years for mayors to obtain a full departmental rank position. The results of column (5) in Table 3 suggest that the effect of the mayor obtaining a full departmental rank position one year earlier on confirmed cases was − 0.37% (59 years old), − 0.46% (53 years old), and − 1.12% (34 years old), respectively. In addition, according to the results of column (4), the pandemic control effect of the mayor can be calculated for different provincial governor promotion speeds and Wuhan’s Baidu migration index. When the promotion speed of the provincial governor took the maximum value and the Baidu migration index for Wuhan City took the average value, the effect of the mayor getting a full departmental rank position one year earlier on confirmed cases was − 1.42% (59 years old), − 1.76% (53 years old), and − 4.27% (34 years old), respectively.

Furthermore, the results of columns (3) and (5) in Table 3 show that when the mayor’s promotion speed is at an average level, the increase in population flowing in from Wuhan city does not cause the increase in confirmed cases (β = − 3.589, P < 0.01; β = − 0.838, P < 0.01). When the Wuhan’s Baidu migration index increased by 0.01, the effect of the mayor with maximum, mean, and minimum promotion speed on confirmed cases was − 4.96%, − 0.82%, and 0.06%, respectively, which indicates that mayors with different promotion speed differed in controlling imported cases, and the mayors with faster promotion speed would be able to avoid the spread of the pandemic caused by imported cases.

### Provincial governor’s effect on the pandemic control

The results of columns (3) and (5) in Table 3 show that the provincial governor’s promotion speed has a significant positive effect on confirmed cases when the mayor’s promotion speed is averaged (β = 5.961, p < 0.1; β = 9.930, p < 0.01). According to the results of column (4), the pandemic control effect of the  provincial governor can be calculated for different mayor’s promotion speeds. When the mayor’s promotion speed took the minimum value, the effect of the  provincial governor’s promotion speed on confirmed cases was positive (β = 15.868, P < 0.01). The effect of the provincial governor’s promotion speed on confirmed cases was negative (β = − 17.332, P < 0.01) when the mayor’s promotion speed was taken as the maximum value. As shown in Table 1, the minimum, mean, and maximum values of the promotion speed of provincial governors are 0.017, 0.019, and 0.030, respectively, corresponding to the ages of 66, 57, and 33 years for provincial governors to obtain a full ministerial rank position. Thus, when the promotion speed of the mayor took the maximum value, the effect of the provincial governor obtaining a full ministerial rank position one year earlier on confirmed cases was − 0.40% (66 years old), − 0.54% (57 years old), and − 1.63% (33 years old), respectively. Combining the above results, hypothesis 1 was validated.

### Heterogeneity test of mayor's effect on the pandemic control

The results of column (6) in Table 3 further show the differences in the pandemic control effects of mayors with different characteristics. The results of column (6) in Table 3 show that the interaction between mayor’s promotion speed and undergraduate major type is significantly negative (β = − 0.947, p < 0.01), the interaction between mayor’s promotion speed and postgraduate degree type is significantly negative (β = − 0.629, p < 0.01), and the interaction between mayor’s promotion speed and title is significantly negative (β = − 6.671, p < 0.01). To obtain more accurate estimation results, Eq. (8) was used for the calculation. The calculation results showed that the regression coefficient of the interaction between mayor’s promotion speed and undergraduate major type was 0.6121, which indicates that mayors with science and engineering backgrounds could reduce the number of confirmed cases by 61.21% on average compared to those with a liberal arts background. The regression coefficient of the interaction between mayor’s promotion speed and postgraduate degree type was 0.4669, which indicates that mayors with a full-time postgraduate degree background could reduce the number of confirmed cases by 46.69% on average than those with part-time postgraduate degree backgrounds. The regression coefficient of the interaction between mayor’s promotion speed and title was 0.9987%, which indicates that mayors with titles could reduce the number of confirmed cases by 99.87% on average than those without titles. These results showed that mayors with different characteristics had different effects on the pandemic control, so hypothesis 2 was validated.

### Robustness test

Table 4 reports the results for replacing the core explanatory variables. Comparing the results of columns (1) to (3) in Table 4 and columns (4) to (6) in Table 3, it can be seen that the regression coefficients and statistical significance of the control variables are minimally changed after replacing the core explanatory variables. The results of column (2) in Table 4 show that the mayor’s real age has a significant positive effect on confirmed cases (β = 0.008, p < 0.01) when the provincial governor’s real age and Wuhan’s Baidu migration index are taken as the mean. When the mayor’s real age is averaged, the  provincial governor’s real age has a significant negative effect on confirmed cases (β = − 0.005, P < 0.01). The provincial governor’s real age significantly positively affects confirmed cases only when the mayor’s real age is less than the mean. Secondly, the results of column (2) in Table 4 suggest that the increase of the inflow population from Wuhan does not cause the increase in confirmed cases when the mayor’s real age is at the average level (β = − 0.814, p < 0.01). Finally, the results of column (3) in Table 4 show that the regression coefficients of the interaction between the mayor’s real age and undergraduate major type, graduate degree type, and title is all significantly negative at the 1% level, which is consistent with the results of column (6) in Table 3.

**Table 4:** Robustness test results for replacing core explanatory variables

Variables/counterpart questions	(1)	(2)	(3)
	FGLS	FGLS_para	FGLS_para*
Mayor’s real age	− 0.324***	0.008***	0.009***
	(0.003)	(0.00008)	(0.00010)
Provincial governor’s real age	− 0.298***	− 0.005***	− 0.005***
	(0.003)	(0.00015)	(0.00017)
Baidu migration index for Wuhan	− 10.170***	− 0.814***	− 0.812***
	(0.057)	(0.005)	(0.005)
Interaction between mayor’s age and Baidu migration index for Wuhan	0.179***		
	(0.001)		
Interaction between mayor’s age and provincial governor’s age	0.006***		
	(0.00005)		
Residents’ awareness of epidemic prevention and control	0.112***	0.112***	0.111***
	(0.00008)	(0.00008)	(0.00007)
Baidu migration scale index	− 0.060***	− 0.060***	− 0.061***
	(0.0002)	(0.0002)	(0.0002)
Number of population is counted by logarithms	0.612***	0.612***	0.608***
	(0.006)	(0.006)	(0.006)
Is the city in the surrounding provinces of Hubei Province (Yes = 1)	0.741***	0.741***	0.737***
	(0.007)	(0.007)	(0.007)
Is the city in Hubei Province (Yes = 1)	3.551***	3.551***	3.532***
	(0.035)	(0.035)	(0.035)
Time trend	0.022***	0.022***	0.022***
	(0.00018)	(0.00018)	(0.00019)
Centering the interaction between mayor’s real age and Baidu migration index for Wuhan		0.179***	0.179***
		(0.00103)	(0.00092)
Centering the interaction between mayor’s real age and provincial governor’s age		0.006***	0.006***
		(0.00005)	(0.00006)
Interaction between mayor’s real age and undergraduate major type			− 0.00037***
			(0.000008)
Interaction between mayor’s real age and graduate degree type			− 0.00028***
			(0.000014)
Interaction between mayor’s real age and title			− 0.002***
			(0.000019)
_cons	14.670***	− 2.680***	− 2.673***
	(0.140)	(0.0283)	(0.0327)
*N*	29,862	29,862	29,862

Columns (1)–(3) are the result of replacing the variable of promotion speed with the real age. Standard errors are reported in parentheses

*p < 0.1; **p < 0.05; ***p < 0.01

## Discussion

Inspired by the principal-agent relationship between the central government and local officials in the theory of official promotion tournament, this study holds that the accountability system implemented in China’s pandemic prevention and control has strengthened the positive behaviors of local officials. To validate this point, this study constructs the promotion gain and accountability cost of officials by using the promotion speed indicator, and analyzes officials' behavioral choices in pandemic prevention and control from a utility maximization perspective.

Some scholars have explained the reasons for China’s success in combating the pandemic. He et al. [5] conclude China’s public crisis management pattern at the institutional, strategic, and operational levels. They argue that the unique political system, resource mobilization, community isolation, and official incentives play a great role in pandemic prevention and control. Mei [27] argues that the policy mix comprised traditional measures, i.e., strict community lockdown, cross-jurisdictional mobilization of resources, and officials’ sanctions, contributed to the effectiveness of pandemic control in China. Zhang et al. [28] suggest that China’s human mobility restriction policies have effectively controlled the pandemic’s spread.

In addition, Ning et al. [29], Jing [30] argue that clear accountability in crisis management is the main determinant of the state’s ability to act decisively. Moreover, He and Guo [31] show that ancient officials played an important role in epidemic prevention and control, and their work mainly included spreading epidemic prevention methods, inspecting epidemic situations on the spot, and treating patients. Mu and De Jong [32] show that accountability has long-term and short-term impacts on officials. The most surprising thing is that local officials who passively fought against the epidemic in ancient China were held accountable. For example, in the fifth year of Jingtai in the Ming Dynasty (1454), Shi Li, the governor of Huguang region, was severely reprimanded by the emperor for his passive prevention and control of the epidemic. These studies show that officials and official accountability play an important role in epidemic prevention and control. However, Wang et al. [33] show that the punishments of China’s official accountability system are too severe, which may lead to adverse selection of officials for fear of being held accountable. It is because severe punishments could reduce their motivation and influence their decision-making [34, 35]. The results of this study suggest that the accountability system motivates local officials to behave positively in terms of pandemic prevention and control. Although this coercive form of motivation may put more stress on officials and be detrimental to their psychological well-being, it has worked in emergency situations.

This study also found a positive correlation between the inflow of population from Wuhan city and local confirmed cases, which suggests that restricting the outflow of population from Wuhan city, i.e., the lockdown of Wuhan city, is effective in the containment of the spread of cases. This finding is consistent with Fang et al. [36]. Another finding of this study is that the intensity of intra-city migration shows a negative correlation with local confirmed cases, which is contrary to Zhang et al. [28]. We explain it from the characteristics of population movement and the COVID-19 pandemic during the Spring Festival travel season. From January 1 to January 22, 2020, the population movement was at its peak, but the number of confirmed cases was small. After January 22, the number of confirmed cases increased greatly. Due to the end of the Spring Festival travel rush and the adoption of pandemic prevention and control measures in all regions, the population movement was small. Therefore, the relationship between population movement and the number of confirmed cases shows a negative correlation. Notably, this study has not yielded a causal relationship between restricting population migration and the spread of confirmed cases because it only takes inter- and intra-city population migration as control variables and does not use causal inference for the research.

Despite our efforts to improve the study, there are still shortcomings in the model setting due to data limitations. The core explanatory variables do not vary over time, making it impossible to control the city fixed effects in the model. Nevertheless, this study holds that the promotion speed variable has good externalities when it is used to explain the number of confirmed cases. In addition, we include effective control variables in the model, which could alleviate the problem of omitted variables due to the no-inclusion of city fixed effects. If better data are available in the future, more sophisticated models could be used for more in-depth research. For example, suppose indicators of local officials’ inputs to the pandemic were available. In that case, the number of confirmed cases could be used to measure the output of local officials and to study the efficiency of pandemic prevention and control by officials in different regions.

## Conclusions

This study shows that the provincial governor’s and mayor’s promotion speed has a significant negative effect on the number of confirmed cases. Mayors with different promotion speeds differed significantly in controlling imported cases. There is a significant interaction between the promotion speed of provincial governors and mayors, which indicates that the provincial governor and the mayor have a coordinated effect on pandemic prevention and control. In addition, the effect of the provincial governor’s promotion speed on the number of confirmed cases depends on the mayor’s promotion speed, which implies that although both provincial governors and mayors play a role in preventing and controlling the pandemic, the mayor’s role is dominant. Moreover, Mayors with different characteristics differed significantly in pandemic prevention and control. Mayors with full-time postgraduate education, titles, and majors in science and engineering have a greater control effect on the number of confirmed cases. Although the findings are from a case study of China, the conclusions have implications for the global fight against the pandemic. Especially in centralized countries, adopting a strict accountability system could be effective in the pandemic containment.

## Data Availability

The datasets used or analyzed during the current study are available from: https://doi.org/10.6084/m9.figshare.17292578.v1. The code used during the current study are available from: https://doi.org/10.6084/m9.figshare.17292593.v1

## Appendix

*Dependent variable* We choose the number of confirmed cases to measure the degree of pandemic control. There are two main reasons for choosing this proxy variable. Firstly, although the Chinese Health Commission published the data of confirmed cases, newly confirmed cases, death cases, newly dead cases, and cured cases, the use of newly confirmed case variable would result in many zero values in the data sample because China had zero newly confirmed cases in 28 provinces and cities on March 7, 2020. Although highly infectious, the lethality of novel coronavirus is low, and using the death cases variable would also result in many zero values in the data sample. Therefore, the data variance of the newly confirmed and death cases is small, and it is difficult to use these variables to get significant results. Second, using the confirmed cases variable could intuitively explain the pandemic control effect of local officials. In the variable processing, considering a small number of zero values in the confirmed case variable data, we add one and then take the logarithm. Since the mean and maximum values of the variables are 11.83 and 3518.24, respectively, this treatment does not change the conclusions of this study.

*Independent variables* We assume that the main subjects in pandemic prevention and control are local officials and residents, and other factors affecting the pandemic are mainly population movements and imported cases. Zhang et al. [28] show that human mobility restrictions in China effectively control the pandemic by reducing the intensity of inter-city and intra-city migration, and the pandemic control effect of mobility restrictions policies is better in areas where residents have higher epidemic prevention and control awareness. Therefore, to better estimate the control effect of local officials on the number of confirmed cases, we control for differences in residents’ awareness of epidemic prevention and control, population movement, imported cases in different cities.

In the measurement of residents’ awareness of epidemic prevention and control, Zhang et al. [28] use the severity of the 2003 SARS as a proxy variable of residents’ awareness of epidemic prevention and control, and Gallè [37] shows that higher levels of knowledge have a higher awareness of pandemic prevention. However, these proxy variables do not reflect the behavior of residents in the process of epidemic prevention and control. Mu and De Jong [32] show that cognitive biases lead to strategic behavior. Therefore, this study chooses Baidu’s overall search index of COVID-19-related terms as the proxy variable of residents’ awareness of epidemic prevention and control. In the measurement of population movements, referring to Zhang et al. [28], we use the Baidu migration scale index as the proxy variable of population movement. In the measurement of imported cases, since five million people left Wuhan before the city was locked down. Therefore, we use the Baidu migration index for Wuhan to track the inflow population from Wuhan in each city during the Chinese Spring Festival travel season.

Furthermore, assuming that mayors affect the number of confirmed cases, it cannot be excluded that provincial governors also have this effect. Therefore, we control the  provincial governors’ effect in different cities. The provincial governors in this paper include the governors of provinces and autonomous regions. In addition, the number of confirmed cases during the study period exhibits a gradient decline from Hubei province to the surrounding areas, with the highest number of confirmed cases in cities within Hubei province, followed by cities in the surrounding provinces of Hubei province, and finally other cities. Therefore, this study uses the dummy variable method to control the spatial effect of confirmed cases.

## Abbreviations

COVID-19: Coronavirus disease 2019
PCSE: Panel-corrected standard error
FGLS: Feasible generalized least squares
